# Infrared thermography as a non-invasive method for evaluating stress in lactating dairy cows during isolation challenges

**DOI:** 10.3389/fvets.2023.1236668

**Published:** 2023-09-06

**Authors:** Madalina Mincu, Ioana Nicolae, Dinu Gavojdian

**Affiliations:** Laboratory of Cattle Production Systems, Research and Development Institute for Bovine, Balotesti, Romania

**Keywords:** animal welfare, animal-based indicators, dairy cattle, infrared thermography, stress response

## Abstract

The overall objective of the current data report was to evaluate and test the feasibility of using infrared thermography (IRT) as a non-invasive method for measuring stress signs in lactating dairy cows during short negative challenges, such as visual isolation from herd-mates. The study was carried out at the Experimental Farm of the Research and Development Institute for Bovine Romania, on 20 Holstein-Friesian lactating multiparous dairy cows, between August and September 2022. Cows were housed in two identical tied stanchion barns (170/85 cm), and were isolated individually from the herd for 240 min post-morning milking. Our results shown significant (*p* ≤ 0.05) rises for both orbital and nasal IRT temperatures following the isolation challenge, suggesting that such approaches could represent adequate tools for assessing social stress in cattle. Overall, current results are in accordance with previous studies which validated both eye and nasal regions as IRT thermal windows for studying the effects of painful and negative contexts on stress response in farmed ruminants, while considering the stress-induced hyperthermia as an integral part of the physiological response to negative stimuli, as well as the current limitations that this tool faces.

## Introduction

Precision livestock farming technologies (PLFs) have been profoundly integrated with farming during the last decade in order to monitor production and reproduction levels, as well as to detect health problems in dairy cattle (1, 2). As a consequence, the use of infrared thermography (IRT) to improve health monitoring and early detection of disease in cattle has gained interest (3). IRT tools were found to be cost- and time effective, reliable as well as non-invasive, while showing great potential for remote sensing and automatization in monitoring early indicators for health abnormalities in dairy cattle, being previously validated for mastitis and lameness detection (4, 5). Moreover, several recent studies had found IRT as a feasible tool to evaluate thermal stress (6–8) and reproduction (9, 10) in cattle. Additionally, both putatively negative and positive emotions have been shown to induce physiological responses in cattle, that allows indirect assessment of the sympathetic and parasympathetic activity via the alterations in temperature of different body areas, caused by phenomena such as stress-induced hyperthermia (11). Such thermal responses to stress and the associated factors that modulate them, have been of great interest among scientists to determine the welfare of the animals, since it is considered that variations in IRT temperature are a reliable and sensitive measure to determine the stress degree perceived by the animal (12).

However, previous research published on cattle commonly involved IRT studies during veterinary painful procedures (e.g., disbudding, castration), or animals with impaired health (e.g., mastitis, lameness, pneumonia), leading to difficulties in separating the effects of fear from the actual response to pain. For instance, following castration, the IRT eye temperature decreased and then increased, compared to baseline values, in male calves that were not given anesthesia, while calves with local anesthesia showed solely an increase in eye temperature (13). These results suggest that the initial drop in the eye temperature, followed by an abrupt increase, is indicative of acute pain, whereas an increase only could be attributed to fear alone. With the author concluding that sudden IRT eye temperature changes might represent a suitable indicator of acute pain in cattle. So far, one individual cattle study explored changes in IRT eye temperature during positive situations, reporting a slight increase during feeding time (14). Correspondingly, Mincu et al. (15) found a slight increase for both eye and nasal IRT temperature in dairy water buffalo pre- and post-milking, with nervous and temperamental buffalo cows expressing higher IRT readings post-milking, compared to calm animals.

Up-to-date results on using IRT tools suggest that measuring thermal windows can provide a nonspecific indication of arousal states in cattle. However, as outlined by previous authors (5, 16), a great series of factors can affect IRT readings and therefore lead to biases, such as equipment settings, distance and angle, and environmental conditions (e.g., temperature, humidity, dust particles, sunlight and air-currents), as a result, procurement and interpretation of these measures requires care and an integrated approach.

It was shown that cattle are highly gregarious, forming complex long-lasting social relationships and are strongly motivated for social contact (17, 18), with social isolation inducing significant negative behavioral and physiological responses. In addition, individuals vary in how well they cope with stressors and challenges (19–21), however, responses to social stressors have focused almost exclusively on competition for resources (22). Throughout a typical production cycle, dairy cows are facing a series of stressful challenges, such as separation from calf, frequent regroupings, isolation from herd-mates (e.g., for insemination, gestation check-ups, housing in sickness pens, drying off). Limited research has been undertaken to validate the use of non-invasive tools, such as IRT measures, for assessing social stress response of adult cattle (23), especially under typical commercial settings and contexts.

The overall objective of the current data report was to evaluate and test the feasibility of using infrared thermography as a non-invasive method for measuring stress signs in lactating dairy cows during short negative challenges, such as visual isolation from herd-mates.

## Materials and methods

The study was carried out at the Experimental Farm of the Research and Development Institute for Bovine in Balotesti - Romania, on 20 Holstein-Friesian lactating multiparous dairy cows (parities II and III, 40 to 120 days in milk), between August and September 2022. Cows from the experimental herd were housed in two identical tied stanchion barns (170/85 cm), and were isolated individually from the herd for 240 min post-morning milking (starting at 07:00 AM). All cows received 3 kg of concentrates 1 h before the commencement of the experimental trial, and remained in their stall, while their herd-mates were allowed access to a nearby paddock in the close proximity of the barn. Therefore, cows were just visually isolated from the herd, while they could hear and communicate vocally with the other cows. During the negative challenge, a series of factors such as frustration build-up caused by lack of exercise and disruption in the cows daily routines, the circadian rhythm of digestive processes and huger caused by fodder-deprivation could have played important roles in the affective response of the animals, as a consequence, the IRT reading values cannot be attributed solely to the social isolation alone. Throughout the isolation, cows had *ad libitum* access to water throughout individual drinkers and fresh wheat straw bedding for animal comfort. During the isolation challenge, cows did not receive any fodder inside the barn, in order for the feeding not to hinder with the IRT readings.

**Figure 1:**
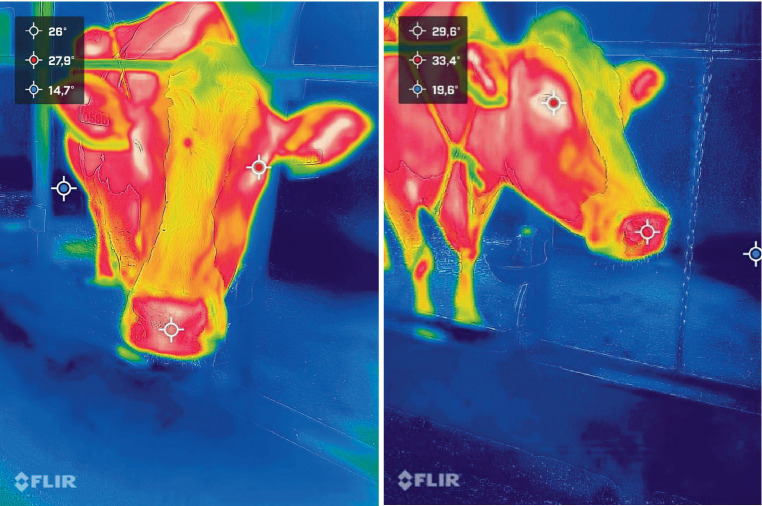
IRT readings for *regio orbitalis* and *regio nasalis* in a dairy cow during experiments, front (left photo) and lateral view (right photo).

IRT readings were taken using two FLIR ONE Pro LT mobile cameras (19,200-pixel resolution, temperature range −20° to 400°C) and FLIR Systems INC© image processing software. Temperature measuring points were the lacrimal caruncle of the eye in the orbital region (*regio orbitalis*) and at the nasal region (*regio nasalis*), which had been previously validated as thermal windows for cattle and water-buffalo (24), with the IRT pictures being taken (x2/animal/region) from a 0.8–1.2 m distance, and an angle of 90° (Figure 1), following the recommendations of Vardasca et al. (25). The three measuring times were as follows: I – pre-isolation, baseline data (0 min); II – 120 min post-isolation from herd-mates; III – 240 min post-isolation from herd-mates.

The temperature inside the barn at the beginning of each experimental day was on average of 21.83°C, with limits ranging between 16.4 and 24.6°C, a relative humidity between 64 and 78%, air speed of <1 m/s, with no direct sun exposure of the animals during the IRT readings.

Using descriptive statistics, we computed the following parameters for the raw data: mean, standard error of the mean (SEM), standard deviation (SD), coefficient of variation (CV), minimum and maximum values, and the first quartile (Q_1_). Comparisons between the 3 time points were carried out using the nonparametric Mann–Whitney U test, all statistical inferences were carried out using Minitab17 software (Minitab LLC®) and decisions about the acceptance or rejection of the statistical hypothesis were made at the 0.05 level of significance.

## Results

To the best of our knowledge, this study is the first to follow changes in IRT temperature at both eye and nose in multiparous lactating dairy cattle, following a visual isolation from herd-mates, while replicating a typical context for the category. An initial dataset of 240 IRT readings was employed, as follows: 3 IRT readings (0 min post-isolation, baseline values; 120 min post-isolation; 240 min post-isolation); 2 regions of interest (*regio nasalis* and *regio orbitalis*); with two IRT readings/animal/time-frame.

**Figure 2:**
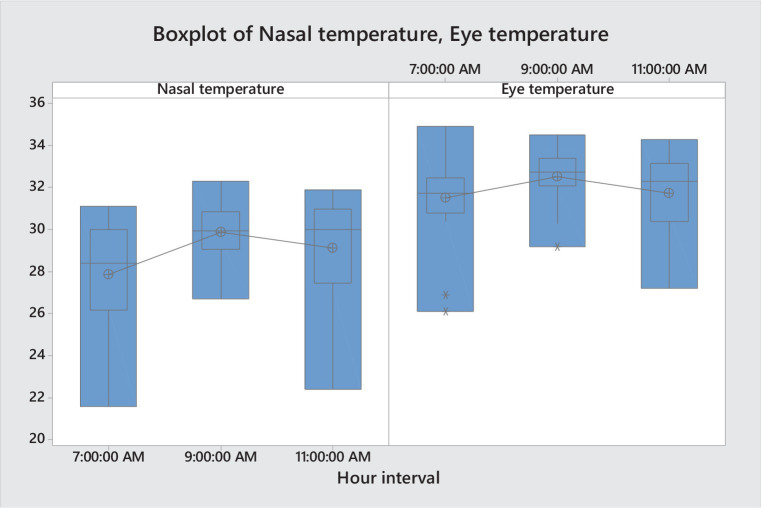
Boxplots of both nasal and ocular IRT temperature for the three-time intervals.

The nasal IRT temperature of cows increased significantly (*p* ≤ 0.005), from the baseline of 27.86 ± 0.54°C to 29.87 ± 0.32°C at 120 min post-isolation, then slightly decreased (*p* > 0.588) at the 3rd reading (240 min post-isolation), to 29.13 ± 0.53°C (Table 1; Figure 2). Although, differences between the IRT baseline temperatures and those registered at the end of the isolation trial were not significant (*p* > 0.05), a tendency towards significance was observed (*p* ≤ 0.069). Contrary to our findings, Proctor and Carder (26, 27) found the nasal IRT temperatures to decrease in both positive and negative situations in cattle. However, these authors used mild positive (feed anticipation) and negative feed related contexts (receiving low quality fodder), while in the current study the cows were faced with a putatively more aversive challenge (18, 28, 29).

**Table 1:** Descriptive statistics of nasal and ocular infrared thermography (IRT) data of dairy cattle at 0 h-, 2 h- and 4 h post-isolation challenge.

Variable	Mean ± SEM	SD	CV	Minimum	Maximum	Q1
IRT nasal region at 0 h [°C]	27.86 ± 0.546	2.44	8.76	21.60	31.10	26.17
IRT nasal region at 2 h [°C]	29.87 ± 0.329	1.47	4.93	26.70	32.30	29.07
IRT nasal region at 4 h [°C]	29.13 ± 0.533	2.38	8.19	22.40	31.90	27.45
Differences 0 versus 2 h		*p* = 0.0055, **				
Differences 0 versus 4 h		*p* = 0.0698, NS				
Differences 2 versus 4 h		*p* = 0.5884, NS				
IRT ocular region at 0 h [°C]	31.51 ± 0.459	2.05	6.51	26.10	34.90	30.80
IRT ocular region at 2 h [°C]	32.54 ± 0.295	1.31	4.05	29.20	34.50	32.10
IRT ocular region at 4 h [°C]	31.74 ± 0.449	2.00	6.33	27.20	34.30	30.40
Differences 0 versus 2 h		*p* = 0.0482, *				
Differences 0 versus 4 h		*p* = 0.4902, NS				
Differences 2 versus 4 h		*p* = 0.2180, NS				

NS, *p* > 0.05; *p ≤ 0.05; **p ≤ 0.01.

The ocular IRT temperature followed a similar pattern to that of the nasal IRT during the isolation challenge. With the ocular IRT temperature increasing significantly (*p* ≤ 0.048) from the baseline value pre-isolation of 31.51 ± 0.45°C, to 32.54 ± 0.29°C at 120 min post-isolation, registering a slight decrease at 240 min post-isolation (*p* > 0.218), to 31.74 ± 0.44°C. The ocular IRT baseline temperature and that registered at the end of the isolation trial was similar (*p* > 0.490), with a slight increase of 0.23°C at the end of the negative challenge. The lower sensitivity found for the IRT ocular temperature changes throughout the 240 min of the isolation challenge, when compared to nasal IRT temperature, could be attributed to physiology of the species, being previously demonstrated (13, 30) that the eye peripheral temperature in cattle quickly decreases after a painful procedure and then increase over baseline values for 15–20 min after the procedure. Such IRT ocular fluctuations have been previously detected at maximum 150 min post hot-iron disbudding in cattle (31), unlike the current study, which challenged the cows for 240 min.

The current pilot study is not without limitations, such as the relative low number of experimental animals, which might have contributed to a lower statistical sensitivity. Moreover, cattle as a species were intensively selected throughout domestication for tameness and docility, which has led to behavioral plasticity and adaptation to current dairy farming practices. In addition, given that the research herd consisted out of multiparous cows, a degree of habituation of the animals to isolation can be assumed. Another potential bias in interpreting the current data is the learned helplessness response, resulting in cows abandoning their attempts to evade the negative challenge due to a perceived lack of control (32), which however, does not translate into the event being perceived as neutral by the animal.

Moreover, the absence of a control group represents a significant limitation of the current pilot-trial, considering that the IRT readings could have been influenced by confounding factors such as behavioral changes associated with time of isolation, as well as potentially by other factors.

Furthermore, for the current trial we used affordable IRT cameras, previously validated for on-farm usage, with lower resolution than the state-of-the-art equipment available, which would increase camera and data sensitivity, while having the significantly higher costs associated drawback.

Overall, current results are in accordance with previous studies which validated both eye and nasal regions as IRT thermal windows for studying the effects of painful and negative contexts on stress response in farmed ruminants, while considering the stress-induced hyperthermia as an integral part of the physiological response to negative stimuli, as well as the current limitations that this tool faces. Moreover, compared to other assessment methods, IRT has the advantage of being non-invasive, while allowing use without the risks of influencing animal behavior or stress physiological responses.

For our future endeavors, to overcome the strong limitations that the current pilot-study faces, we plan to include and use additional sensors to study animal behavior response to negative challenges, such as heart monitors and accelerometers.

The current findings, suggest that changes in both orbital and nasal IRT temperatures following isolation from herd-mates could represent adequate tools for assessing social stress in cattle. Further research needs to be conducted in order to discern between putatively positive and negative contexts, on larger sets of animals and with different degrees of habituation levels to challenges. Our findings support previous research which suggest that there is potential for IRT measurements to be used as non-invasive animal-based indicators of stress in cattle.

## Data availability statement

The original contributions presented in the study are included in the article/Supplementary material, further inquiries can be directed to the corresponding author.

## Ethics statement

Current experiment was approved by an internal review committee from the Research and Development Institute for Bovine (approval code PN-III-P1-1.1-TE-2021-0027, issued on 11.07.2022). The study was conducted in accordance with the local legislation and institutional requirements.

## Author contributions

DG conceived the study and obtained funding. MM, IN, and DG performed animal trials, data collection, and data interpretation. MM wrote the initial manuscript. IN and DG performed manuscript revision. All authors contributed to the article and approved the submitted version.

## Funding

This work was supported by a grant of the Ministry of Research, Innovation and Digitization, CNCS - UEFISCDI, project number PN-III-P1-1.1-TE-2021-0027, within PNCDI III.

## Conflict of interest

The authors declare that the research was conducted in the absence of any commercial or financial relationships that could be construed as a potential conflict of interest.

## Publisher’s note

All claims expressed in this article are solely those of the authors and do not necessarily represent those of their affiliated organizations, or those of the publisher, the editors and the reviewers. Any product that may be evaluated in this article, or claim that may be made by its manufacturer, is not guaranteed or endorsed by the publisher.
